# 

**Published:** 1900-04-07

**Authors:** 


### The Hospital

A JOURNAL OF
THE MEDICAL SCIENCES AND HOSPITAL ADMINISTRATION,
INCLUDING
A SPECIAL SECTION FOR NURSES.

IN TWO VOLUMES ANNUALLY.

EDITED BY
SIR HENRY BURDETT, K.C.B.,
AND
SOLOMON C. SMITH, M.D.

LIBRARY

SURGEON GENERAL'S OFFICE

FEB. 11-1901

168991.

VOLUME XXVIII.

April to September, 1900.

London:
PUBLISHED BY THE SCIENTIFIC PRESS (LIMITED), AT THE HOSPITAL BUILDING,
28 & 29, SOUTHAMPTON STREET, STRAND, W.C.

MDCCCC.

HO69X

### INDEX TO VOL. XXVIII., 1900.

*** Articles under the general heading *Hospital Clinics and Medical Progress* are distinguished by the initial (C.) for *Hospital Clinics*, and by (P.) for the rest of the series. (W.) distinguishes the *Institutional Workshop* articles, and (A.) the *Annotations*.

Abdominal Section, The Mortality of (C.), 268
Acne (A.) 255
Acne, Treatment of (C.), 435
Addison's Disease (P.), 152
Advertising (A.), 63
Advertising Dentists, 179
Aix Douche Massage, The (C.), 116
Alcohol, Effects of, on the Brain (P.), 337
Alcohol, Toxic Action of (P.), 385
Alopecia (P.), 272
Ambulance Arrangements (C.), 400
Ambulance Tongas from India for South Africa (W.), 326
Anæmia, Pernicious (P.), 136
Anæsthetic, Choice of an (P.), 256
Anæsthetics, Progress in (P), 256
Anastomosis, Intestinal (P.), 437
Anderson *v.* Moeller, 112
Antiseptics (P.), 205
Antiseptics in Minor Surgery (C.), 115
Anti-Typhoid Inoculation (C.), 218, 268, 436
Apomorphine (C.), 49
Apothecaries, The Society of, of London (A.), 21[illegible]
Appendicitis (C.), 185
Appendicitis, Association of, with Disease of the Right Adnexa (P.), 319
Appendicitis, Operation in (C.), 399
Architects and Modern Hospitals (W.), 39
Army Doctors in the Future, 295
Army Medical Department, The (A.), 264
Army Medical Service, The Future of the (C.), 351
Arsenical Phosphate of Soda, 197
Arthritis, Rheumatoid (C.), 133
Artificial Anus (P.), 437
Artificial Serum in Puerperal Septicæmia (A.), 147
Assam, Dispensaries of (W.), 59
Automobile, The, in Country Practice (C.), 366
Bacillus Tuberculus, Thermal Death Point of (P.), 287
Backache (C.), 68
Bacteria and Digestion (P.), 16
Bacteria and Sewage, 78
Bacteriuria (P.), 137
Bacteriology, Progress in (P.), 15, 287
Baths and Waters, The Treatment of Disease by, 5
Beggar, How to Train the (A.), 97
Bicycle, To Perdition on a, 55
Bladder, Hernia of the (P.), 286
Bladder and Prostate Gland, Diseases of the (P.), 85, 103
Bladder and Rectum, Wound of the (P.), 121
Bloemfontein Scandals, The (A.), 233
Blood, Diseases of the (P.), 136, 152
Blood-stained Pillow, The (A.), 233
Boer and British Sanitation (C.), 317
Book World (*Books, Magazines, and Pamphlets Reviewed*)
Austen. *See Ross*
Bacon's War Atlas, 88
Broadbent. Heart Disease, 18
Burdett's Hospitals and Charities, 274 and *see p.* 360
Buxton. Anæsthetics, Their Uses and Administration, 322
Chapman. Heart Disease in Childhood and Youth, 174
Coats (revised by Sutherland). Manual of Pathology, 18
Corbet. Lunacy: The Rising Tide, 140
Dührssen (trans. by Taylor and Edge). Manual of Gynæcological Practice, 122
Edge. *See Dührssen*
Elder. The Physical Basis of Memory, 174
*Excelsior*, 1899-1900, 356
Haig. Diet and Food, 224
Hall. Golden Rules of Physiology, 88
Halliburton. Handbook of Physiology (Kirke's Handbooks), 322
Hartridge. Golden Rules of Ophthalmic Practice, 406
Hillier. Tuberculosis: Its Nature, Prevention, and Treatment, 322
Hobhouse. Health Abroad, 54
Hood. The Early Treatment of Appendicitis, 406
Jardine. Practical Text-book of Midwifery, 372
John Bull's Trip to Paris, 274
Keith. Plea for a Simpler Life, 156
Lucas. Nordrach at Home, 224
Lutaud. Manuel complet de Gynécologie médicale et chirurgicale, 122
Book World (*continued*)—
Macdonald. The Passmore Edwards Institutions, 140
Macnaughton-Jones. Practical Manual of Diseases of Women, 122
Malarial Fever, Instructions for the Prevention of, 372
Matriculation Directory, The, 88
Maylard. Student's Handbook of the Surgery of the Alimentary Canal, 174
Medical Annual and Practitioners' Index, 18
Norris. Primer of the Physiological Action of Alcohol, 88
Norris and Oliver. System of Diseases of the Eye, 36
Oliver. *See Norris*
Paget. Experiments on Animals, 406
Perkins. Lateral Curvature of the Spine, 140
Richet. Dictionnaire de Physiologie, 54
Robson. Diseases of the Gall Bladder and Bile Ducts, 190
Rogers (edited by). Pye's Surgical Handicraft, 88
Ross and Austen. Report of the Malaria Expedition, 224
Smith. Paralytic Deformities of the Lower Extremities, 190
Smith (Heywood). Practical Gynæcology, 221
Stark. Aids to Pharmacy, 72
Steen. Evolution of Asylum Architecture, 51
Steen. Modern Asylum Construction, 406
Steevens. Things seen, 406
Stevenson. Contagious Ophthalmia, Acute and Chronic, 72
Supplement to the 28th Annual Report of the Local Government Board, 140
Sutherland. *See Coats*
Taylor. *See Dührssen*
Tubby. Appendicitis, 372
Twentieth Century Practice of Medicine, Vol. xix., 356
West. On Granular Kidney and Physiological Albuminuria, 156
Wiechmann. Chemistry: Its Evolution and Achievements, 72
Wilson. Brain and Body, 54
"Boycott" of a Congress, The (A.), 113
Brain Injury, Cases of (P.), 353
Brains and Railway Accidents, 231
Brandy (A.), 131
British Association and Malaria, The, 395
British Medical Association, The (A.), 265
British Medical Association, The Address in Surgery, 300
British Medical Association and the O'Hara Case, The (C.), 366
British Medical Association, The President's Address (C.), 299
British Pharmacopœia, The (A.), 297
Buller (Sir Redvers) and the Royal Army Medical Corps (A.), 379
Bullet Wounds, Some Exploded Theories about (C.), 400
Bullet Wounds of the Spinal Cord (P.), 403
Butchers and Tuberculous Meat (A.), 264, 310
Cacdylic Acid (P.), 273
Cancer (P.), 220
Cancer of the Breast, Expectancy of Life in Cases of (P.), 401
Cancer of the Breast, Some Errors about (C.), 13
Cancer of the Rectum (P.), 384
Cancer of the Stomach (P.), 33
Cerebral Surgery (P.), 353, 369
Cerebro-spinal Meningitis (P.), 368
Certificates (A.), 147
Chemical Tests (in Diseases of the Digestive Organs) (P.), 32
Chemists' Exhibition, The, 229
Child-Worker, The, 323
Chinese Question, The (A.), 181
Chlorosis (P.), 152
Cholelithiasis (P.), 50
Chrome Poisoning (A.), 249
Cigarette Smoking, 329
Circumcision (C.), 284
Cirrhosis, The Treatment of (C.), 66
Civil Dispensaries of the Burma Police, The (W.), 359
Classification in Workhouses (A.), 3
Climate and Clothing, 61
Colic, Biliary (C.), 49
Consultation at the Polyclinic, A (C.), 99
Copper—Is Copper a Poison? (A.) 415
Coroner, The Office of (A.), 63
Cortical Excision for Traumatic Epilepsy (P.), 353
Criminal on the Continent, The, 440
Criminality and Degeneracy (P.), 320
Criminals, Young 191
Crumbs from the Doctor's Table (A.), 347
Cyst, Traumatic Pancreatic (P.), 51
Cystinuria (P.), 137
Cystitis (P.), 85, 385
Deafness, Influence of Heredity on (P.), 86
Death by Starvation (C), 317
Degeneracy (Psychiatry) (P.), 172
Degenerates, General Considerations Regarding (P.), 187
Dermatology, Progress in (P.), 255, 272
Dermoids, Cranial and Spinal (P.), 369
Diabetes (P.), 186
Diabetes, The Cause of (C.), 252
Diabetes, Diet in (C.), 134
Diabetes from Nervous Strain (C.), 185
Digestive System, Diseases of the (P.), 32, 118
Digitalis, How to Give (C.), 382
Doctor and the Nurse, The (A.), 313
Doctor's Pigeons, The (A.), 20
Doctors and the Press (A.), 281
Drawing and Medicine (A.), 3
Dressing a Case (C.), 149
Drill Sergeant in the Board School, The, 37
Drink and Heredity (C.), 335
Drunkenness in the French Army (A.), 165
Dysmenorrhœa, Intermenstrual (P.), 337
Eclampsia (P.), 16, 53
Eclampsia, Clinical Lecture on (C.), 217
Eclampsia, Puerperal (P.), 240
Ectopia Vesicæ (P.), 86
Editor's Letter Box, 76, 109, 127, 161, 195, 241, 261, 310, 360, 375, 411
Electric Trams and Suburban Industries (A.), 378
Endermol (P.), 273
Enteric Fever (P.), 287
Enteric Fever Among Soldiers (C.), 252
Epileptics, The "Colony Treatment" of (C.), 435
"Eternal Vigilance" (C.), 168
Ether (P.), 257
Eucaine Anæsthesia (C.), 13
Exercise, The Abuse of (C.), 201
Expanding Mauser Bullets (C.), 201
Experiments upon Animals (C.), 31
Experiments upon Living Animals (A.), 265
Fæculent Vomiting which is Sometimes Curative (C.), 12
Failure of the Filters, The (A.), 113
Family Periodic Paralysis (P.), 352
Feeble Minded, The Training of the, 123
Feeble Minded, The Welfare of the (A.), 45
Feeding the Navy (A.), 331
Fees for Certificates (A.), 45
Fibroids Complicating Pregnancy (P.), 240
Field Dressings (C.), 400
Fighting a Famine, 373
Food Poisoning, 2
Food Preservatives (C.), 253
Fowls and Diphtheria (A.), 198
Fractures by Torsion (C.), 269
Fractures, Operations in Simple (C.), 151
Fractures, Treatment of, by Movement and Massage (C.), 349
Fractures and Dislocations (P.), 51, 221
Fractures and Dislocations, Lower Extremity (P.), 69, 421
Fractures and Dislocations, Upper Extremity (P.), 68, 403, 420
Gables Hospital, Surbiton, The (W.), 275
Gangrene of the Penis (C.), 185
Gastric Lavage (P.), 32
Genealogical Studies (P.), 70
General Election, The, 429
General Surgery (P.), 51, 68, 205, 221, 403, 420
Genito-Urinary Surgery (P.), 354, 370, 384
Genius and Degeneracy (P.), 304
Glances at the Hospitals (W.), 90, 124, 193, 228, 245, 292, 309, 326, 359, 375, 408, 426, 441
Glover (Dr.) and his Constituents (A.), 63
Glycosuria, Non-Diabetic (C.), 48; (P.), 186
Gonorrhœal Rheumatism (P.), 102
Gout (C.), 13; (P.), 84, 102, 270
Gout and Hay Fever (C.), 253
Gout, Rheumatism, and Rheumatoid Arthritis (C.), 133
Gout, Two Old-fashioned Remedies in (C.), 220
Growth of Boys, The (A.), 79
Gunshot Wounds (C.), 150

Gynæcological Society, The (C.), 82
Gynæcology, Progress in (P.), 103, 138, 319, 337
Hæmorrhoids (P.), 120
Hardy Old Doctor, A (A.), 431
Health of the Navy, The (A.), 347
Heat Stroke (A.), 181
Hemiplegia, A Case of (C.), 99
Hepatic Tuberculosis (P.), 119
Hernia (P.), 286, 303
Hernia, Bilocular (P.), 239
Hernia, Encysted (P.), 254
Hernia, Strangulated, in an Infant (P.), 304
Hernias, Sliding of the Cæcum and Sigmoid Flexure (P.), 254
Heroin, The Therapeutic Value of (P.), 170
History of a Quarrel, The (A.), 164
Holidays, 25, 345
Hospital Construction (W.), 19, 56, 275, 290, 358, 374, 408
Hospital Festivals, Meetings, &c., 20, 41, 74, 93, 107, 126, 142, 158, 175, 194, 211, 226, 242, 276, 293, 327, 341, 411
Hospital Politics and the Central Funds (A.), 297
Hospital Section at the Woman's Exhibition (W.), 161
Hospitals Inquiry, The, 279
Hospitals and Rates (W.), 340
Hot-air Treatment (C.), 351, and *see p.* 375
Hypertrophic Pyloric Stenosis in Infancy (C.), 203
Ice-bag in the Treatment of Pneumonia, 202
Idiocy, Amaurotic Family (P.), 336
Immunity (A.), 248
Imperial Yeomanry Base Hospital at Deelfontein, Notes on the (W.), 425
Imperial Yeomanry Hospital, The (W.), 208, 258, and *see p.* 261
Indigestion, Sir T. Lauder Brunton on (C.), 100
Industrial Lead Poisoning (C.), 285
Infant Symptoms and Modification of Food (C.), 65
Infants, The Artificial Feeding of (C.), 335
Infants, The Over-Feeding of (C.), 29
Insane, The Story of the, from Year to Year (W.), 39, 58, 91, 107, 125, 409, 427, 442
"Insane Persons," "Procedures Relative to the Single Care of" (C.), 2[illegible]7, 283, 315
Insanity, Pathogenesis of (P.), 71
Insanity, Pathology and Treatment of, 232
Insanity, On the Prevention of (C.), 301
Insanity and Race Decay (P.), 52
Insomnia (P.), 318
Insomnia, The Physical Treatment of (C.), 235
Instinct *versus* Discipline, 26
Institutional Treatment of Disease, The, 430
Institutional Questions (W.), 60, 93, 127, 229, 261, 294, 310, 343, 443
Insurance Certificates (A.), 414
International Medical Congress, The, 312
Intestinal Obstruction (P.), 437
Intestines, Surgery of the (P.), 437
Intussusception (P.), 438
Investors and Visionaries (A.), 165
Ipswich Meeting of the B.M.A., 280
Isolation within Isolation Hospitals (C.), 81
Jaundice, A Case of (C.), 99
Joys of a Congressist, The (A.), 331
Kernig's Sign in Meningeal Hæmorrhage (P.), 368
Kidney, Movable (P.), 34
Kidney, Rupture of the (P.), 34
Kidneys, Diseases of the (P.), 137
Labour, Painless (C.), 417
Ladies as Infirmary Visitors (A.), 313
Laryngology, Progress in (P.), 405, 422, 436
Lead Poisoning and Insanity (C.), 352
Lead Poisoning among Women (C.), 237
League of Mercy, The (W.), 306
Legislation by Definition (A.), 113
Leprosy (P.), 272
Lessons from the War (A.), 116
Leukæmia (P.), 136, 152
Light and Vision (C.), 251
Lime-Juice Ration, The, 130
Lithotomy (P.), 85
Liver, The (P.), 118
Liver, Floating (P.), 119
Liver, The Rôle of the (C.), 49
Liver, Rupture of the (C.), 117
London Water Supply, The (C.), 315
Lunacy Scandals (W.), 192
Lunacy "Scandals," The So-called, 263
Lunatic Asylums of India, The (W.), 90
Lunatics, Hopeless, The Private Care of (A.), 397
Lupus (P.), 255
Lupus, Treatment of, by Concentrated Light (C.), 251
Lupus, Treatment of, by X-Rays (C.), 101
Madras, Medical Institutions of the Presidency Town of (W.), 426
Mafeking, 129
Malaria Parasite, The (C.), 117
Manchester, Medical Charities of (W.), 59
Marriage of First Cousins, The, 180
Massage in Recent Fractures (C.), 169
Mastoid Operation in Chronic Otorrhœa (P.), 171
Mastoiditis (P.), 18[illegible]
Measles, Epidemic, 163
Measles, Preliminary Rashes in (C.), 367
Mecca and its Pilgrimage (A.), 131
Medical Ethics in the Sixteenth Century (C.), 253
Medical Fees from Hospital Patients (W.), 175
Medical Inspection of Schools (W.), 259
Medical Profession in the United States, The (C.), 381
Medical Qualifications, 388
Medical Schools, 389
Medical, Surgical, and Hygienic Exhibition, 1900 (W.), 192, 210
Medullated Nerves (P.), 383
Meningitis, The Various Forms of (C.), 167
Metropolitan Asylums Board of London, The, and its Work (W.), 424
Metropolitan Hospital Sunday Fund, 211(W.),307
Middlesex Hospital, New Cancer Wards (W.), 19
Midwives' Bill, The (A.), 45, 233
Midwives, Legislation for (W.), 124, 142, 157, and *see p.* 195, 261
Migraine (P.), 352
Milk Standards (A.), 27
Mind Your Own Business, 213
Mining Accident, A (C.), 83
Modern Elevator, The (A.), 215
Modern Sociology, 37, 55, 89, 106, 123, 191, 323, 357, 373, 407, 440
Mosquito-proof Dwelling-house, A (C.), 117
Mosquitoes Again (C.), 220
Mouldy Stomach, A (C.), 316
Municipal Mothers' Milk (A.), 362
Municipal Sanitary Missionary (A.), 415
Murphy Button (P.), 437
Muscular Rigidity in Acute Peritonitis (C.), 184
Narcolepsy (P.), 337
National Hospital, The, 247
National Hospital for the Paralysed and Epileptic, The (A.), 199; (C.), 324; (W.), 358; (A.), 379
Negro, The (C.), 83
Nervous System, Degeneration in the (P.), 318
Neurology (P.), 302, 318, 336, 352, 368
Neuromata (C.), 384
Neurone, Degeneration of the (P.), 302
New Appliances and Things Medical, 17, 35, 53, 87, 105, 121, 139, 155, 173, 207, 241, 257, 273, 289, 321, 339, 355, 405, 423
Nitrous Oxide (P.), 256
Normal Saline in Septic Cases (C.), 48
Notes and News, 4, 28, 46, 64, 80, 99, 114, 132, 149, 166, 183, 200, 216, 247, 266, 282, 299, 314, 332 348, 364, 380, 398, 416, 432
Notification and Isolation (C.), 183
Obstetrics (P.), 240
Obstetrics, Progress in (P.), 153
Œsophagus, Rupture of the (C.), 316
Old Men's Children (C.), 219
Open-Air "Cures" and Open-Air Work (C.), 367
Open-Air Treatment at Hampstead (A.), 3
Ophthalmology, Progress in (P.), 288
Optic Chiasma (P.), 383
Otitic Abscess of the Brain (P.), 207
Otitic Meningitis (P.), 207
Otitis Media (P.), 154
Otitis Media, Chronic, Ossiculectomy in (P.), 188
Otology, Progress in (P.), 86, 154, 178, 188, 206
Our "Consulting Surgeons," 77
Our Dealings with the Dead (A.), 362
Our Specialists (A.), 347
Ovaritis, Sclero-Cystic (P.), 337
Pancreas, Tumours of the (P.), 51
Paraldehyde, Poisoning by (C.), 435
Pariplegia, Compression (P.), 384
Patella and Olecranon, Fracture of (C.), 201
Pathological Problems of the Day, On Some (C.), 333
Pemphigus (P.), 256
Penal Powers of the General Medical Council (A.), 146
Perforation in Typhoid Fever, The Treatment of (C.), 365
Peritonitis, Septic, The Treatment of (C.), 82
Pharmaceutical Society, The (A.), 431
Phthisis, Drug Treatment in (P.), 170
Phthisis, Surgery in (C.), 236
Physical Education, 145
Physics of Steam Sterilisation (C.), 168
Plague in Europe (A.), 346
Plague in Glasgow (A.), 362, 378
Plague, The Incubation of (C.), 170
"Plague of Women," The, 118
Plague Cases, Accommodation for, in London (A), 396
Plantar Reflex (P.), 383
Pneumococcal Osteo-Arthritis (P.), 239
Political Assassination, 311
Porro's Operation and Cæsarian Section (C.), 284
Potts' Disease, Forcible Correction of Deformity of (P), 222
Practical Departments (W.), 23
Practice by Companies (C.), 237
Practice, Our Field of (A.), 331
Prince of Wales's Hospital Fund (W.), 225
Princess Christian Hospital Train (W.), 141
Proctologist, The (C.), 135
Profession, The, and Midwives (A.), 96
Professional Prospects, 378
Prolapsus Uteri (P.), 139
Prostatectomy and Bottini's Operation (P.), 103
Prostatic Hypertrophy (P.), 354
Ptomaine Poisoning, 296
Public Gardens (A.), 297
Puerperal Fever (C.), 334
Puerperal Fever, Notification of (C.), 417
Puerperal Infection (P.), 271
Puerperal Insanity (P.), 154
Purpura (P.), 102, 270
Pyelonephrosis (P.), 33
Psychiatry, Progress in (P.), 52, 70, 171, 187, 304, 320, 371
Queen Charlotte's Hospital, Improvements at (W.), 56
Quinine and Malaria (C.), 238
Rabies and its Lessons (A.), 215
Radiography, The Errors of (C.), 383
Radio-therapeutics (P.), 273
Railway Carriages and Infection (C.), 419
Rate-Supported Hospitals (A.), 414
Rectum, Cancer of the (P.), 384
Rectum, Imperforate (P.), 119
Rectum, Stricture of, Non-Malignant (P.), 120
Rectum and Anus, Diseases of the (P.), 119
Red Cross, The (C.), 101
Reigate Infectious Diseases Hospital (W.), 56
Removal from the Register (A.), 199
Renal Pelvis, Rupture of the (P.), 35
Resection of Intestine for Gangrenous Hernia (P.), 303
Resorcin (P.), 273
Respiratory System, Diseases of the (P.), 170
Responsibility for Infection (A.), 363
Responsibility for the Sickness amongst our Troops (A.), 214
Rheumatism, Rest in (C), 12
Rheumatism and Gout (P.), 84, 102, (C.), 133, (P.), 204, 238, 270
Right to Die, The, 62
Roosevelt Hospital, The (C.), 68
Rotten Fruit (A.), 131
Royal London Ophthalmic Hospital (W.), 73, 291
Royal Military Benevolent Fund (W.), 306
Rubber Dam in Abdominal Operations, The Use of a (C.), 365
Ruts and Routine (C.), 269
Ryde Hospital, New Hospital for Children (W.), 408
Sanitary Arrangements for Women Workers (A.), 281
Sanitary Authorities, Competing (A.), 233
Sanitary Law, The Enforcement of, 111
Sapodermia (P.), 273
Scarlet Fever (P.), 15
Schools and Measles (C.), 30
Scurvy (P.), 270
Scurvy Among the Troops (C.), 367
Sea and its Dead, The (C.), 150
Seccotine in Surgery (C.), 203
Septic Tank (P.), 338
Serum Therapy in Tuberculous Diseases of the Lungs (P.), 171
Sewage Disposal (P.), 338
Sewage Nuisance, An Indirect (C.), 435
Sexual Function and Insanity (C.), 433
Small-Pox (A.), 249
Small-Pox, Ambulant (C.), 152
Southampton, New Isolation Hospital for (W.), 374, and *see p.* 411
Spastic and Paralytic Deformities, Surgical Treatment of (P.), 223
Speed in Operations (C.), 350
Spinal Caries, Reduction of the Deformity of (P.), 402
Spinal Disease, The Prevention of the Humped Back of (C.), 419
Spinal Fracture and Paraplegia (P.), 402
Spine, Surgery of the (P.), 384, 402
Spines, Carious, Forcible Straightening of (C.), 418
Splenectomy (P.), 50
Spondylitis (P.), 239, 270
Sponge Counting (C.), 83
Standardisation of Drugs, The (A.), 215, and *see* p. 351
State Medicine, Progress in (P), 338, 438
Static Method in X-ray Work (C.), 204
Statistics (C.), 50
Status Epilepticus (P.), 336
Sterility, The Treatment of (P.), 103
Stock Diseases of Humanity, The (A.), 147
Street Cleansing, 44
Stress, Forms of, in the Production of Insanity (P.), 370
Stricture of the Urethra (C.), 183
Student of Medicine, The, 24, 60, 76, 94, 110, 128, 162, 246, 262, 294, 328, 344, 360, 376, 394, 412, 428
Sunstroke (A.), 313
Surgery, Cerebral (P.), 353, 369
Surgery of the Gall-Bladder, Spleen, and Pancreas (P.), 50
Surgery, General (P.), 51, 68, 205, 221, 403, 420

Surgery, Genito-Urinary (P.), 33, 354, 370, 384
Surgery of the Intestines (P.), 437
Surgery, Orthopædic (P.), 222
Surgery of the Spine (P.), 384, 402
Surgery cf the Vermiform Appendix (P.), 14
Suture, Intestinal, Experiments on (P.), 438
Sutures after Abdominal Incisions (O.), 169
Swindling in Life Assurance (O.), 269
Symphysiotomy (P.), 153
Syphilis (P.), 370
Syphilis, The Present-day Treatment cf (O.), 31
Tabes, Gastric Crises of (P.), 336
Tannaform (P.), 272
Tannate of Mercury (O.), 135
Teeth, The Question of (A.), 79
Teeth of the Rising Generation, The (A.), 415
Theft, The Legalisation of, 89
Therapeutical Notes, 16, 105, 121, 173, 189, 207
Thirst, 146
Thrombosis of the Lateral Sinus (P.), 206
To Help Lone, Lorn Women, 357
Truss, How to Measure for a (O.), 284
Tuberculosis (P.), 287
Tuberculosis, Hepatic (P.), 119
Tuberculosis, Prevention of (P.), 438
Tumours of the Breast, Doubtful (O.), 81
Tumours Complicating Pregnancy (P.), 154
Tumours of the Pancreas (P.), 51
Typhoid Fever, The Newer Knowledge on, 1
Typhoid Fever in Paris (A.), 181
Typhoid and Malarial Fever, The Concurrence of (O.), 401
Ulcer, Gastric (P.), 32
Ulceration and Perforation (P.), 437
Umbrellas on the Field cf Battle (A.), 397
Unconventional Inspector, The, 330
University Teaching in London (A.), 248
Ureter, Rupture of the (P.), 35
Ureters, Injury of the (P.), 35
Urinary Antiseptics (P.), 385
Urotropine in Typhoid Fever (O.), 67
Uterine Carcinoma, Treatment of (P.), 319
Uterine Fibroids, The Modern Treatment of (O.), 82
Uterine Fibroids, The Operative Treatment of (P.), 138
Uterine Fibroids, Treatment of (P.), 320
Uterus, Backward Displacement of the (P.), 105
Vaccine Lymphs (P.), 285
Vaginal Hysterectomy for Carcinoma Uteri (P.), 139
Vagrants, 413
Vasectomy (P.), 354
Vermiform Appendix, Surgery of the (P.), 14
*Verulam Review, The*, and the British Medical Association (C.), 101
Vesical Calculi (P.), 384
Vinegar and Alcohol (A.), 79
Vivisection and the Hospitals (A.), 164
Volvulus in Association with Hernia (P.), 286
Vomiting of Pregnancy, The (C.), 271
Waists and Corsets (A ), 431
Wandering Kidney (P.), 370
War and Its Wounded (A.), 198
Warnford Hospital, New Wing (W.), 358
Water Gas (A.), 27
Water for Moving Troops (A.), 396
Water, The Provision of Pure Drinking, for Troops on the March (C.), 350
Weapon, A Dangerous (A.), 97
What the Nations Drink, 407
Where to Vaccinate (A.), 281
Who is to Standardise Drugs (C.), 351
Whooping Cough (P.), 6
Women and Business Life, 106
Women as Organisers, 361
Women and Warfare, 95
Word, A, to Would-be Students on the Course of Study, 387
Word Blindness, Congenital (P.), 368
Working Class Prosperity (A.), 199
Workmen's Compensation Act, The (A.), 363
Wounds and War (A.), 96
X-ray in the Law Courts, The (C.), 134

### THE SPECIAL SECTION FOR NURSES.

Across the Seas, 32, 82, 155, 297
Adelaide District Nurses, 301
Annual Conference of the Matrons' Council, 203
Appointments, 23, 29, 32, 46, 57, 74, 89, 108, 123, 152, 164, 193, 202, 207, 220, 235, 247, 259, 273, 288, 301, 317, 326, 341, 358
Appointments, Minor, 21, 32, 46, 57, 75, 87, 108, 123, 136, 152, 164, 175, 193, 220, 235, 250, 255, 276, 301, 317, 326, 342, 360
Army Nursing Service Bill in the United States, The, 18
Asepsis, On, 81
Association of Asylum Workers—Annual Meeting, The, 97
Book and Its Story, A, 62, 100, 156, 216, 289, 330
Bringing the Sufferers Home, 122
Care of Patients Before and After Operations, On the, 245
Changed Opinion, A, 6
Cold-Water Treatment, A Method of, 33
Colonial Nursing Association, The, 109
Compulsory Registration of Midwives, 229
Consumptive Sanatoria, Bridge of Weir, The, 17
Convalescent Soldiers on Land and Sea, 257
Cookery for Invalids, 61
Co-operation Among Older Nurses, 29
Council Meeting of the Royal British Nurses' Association, 220
Day of Rest for the Metropolitan Nurses, A, 195
Death in Our Ranks, 45, 57, 71, 87, 101, 164, 204, 220, 286, 296
Dispatch of Sixty Nurses to South Africa, 134
District Nursing in the West of Ireland, 7
Duty to the Dying, Our, 96
East-End Mothers' Home, 29
East London Nurses at the Mansion House, 55
Echoes from the Outside World, 13, 36, 47, 60, 73, 86, 99, 112, 124, 139, 153, 166, 181, 194, 206, 218, 234, 248, 260, 271, 285, 300, 313, 327, 343, 357
English Hospital, Haifa, The, 15, 35, 45
Everybody's Opinion, 22, 37, 48, 59, 75, 88, 101, 114, 127, 141, 157, 169, 182, 195, 207, 219, 235, 247, 261, 276, 288, 302, 316, 329, 344, 356
Experiences in a Plague Hospital, 308, 328
Famine Districts in India, Working in the, 352
Fatal Accident to a Sister at the Belgrave Hospital for Children, A, 74
First Aid to Gun Shot Wounds, 81
Functions of the Week, 201, 228
Her Wedding Day, 33
Hints for Nurses, 57
Hospital Ship "Princess of Wales," The, 227
How the Dublin Nurses Saw the Queen, 30
Howard de Walden Nurses' Home and Club, 95
Important to all Trained Nurses, 246
In Memoriam: Elizabeth Grace Hanan, 270
Incident in a Matron's Experience, 358
Interview with Mr. Burdett-Coutts, M.P., 189
Invalided from Bloemfontein, 255
Irish Soldier on the Nurses at the Front, An, 125
Journey from Capetown to Pretoria, 287
Last Duties to Patients, 256
Lectures on Medicine to Nurses, 4, 42, 68, 94, 120, 148, 174, 200, 226, 281, 307, 336
Lectures on Nursing for Probationers, 28, 54, 80, 103, 132, 162, 188, 212, 240, 266, 293, 322, 350
Letter from One of the Army Nurse Martyrs, A, 142
London School Nurses' Society, 204
Male Nurses of the "Maine," The, 87
Medical, Surgical, and Hygienic Exhibition, 138
Miss Florence Nightingale, 95
Modern Aseptic Operation, A, 299
Modern Treatment of Epileptics, The, 83
Netherlands-Indies, Some Hospitals in the, 296, 311, 325
Night Nurses' Wail, The, 57
Notes on Hygiene, 314
Notes on News from the Nursing World, 1, 25, 39, 51, 65, 77, 91, 103, 117, 129, 145, 159, 171, 185, 197, 209, 223, 237, 251, 263, 279, 291, 305, 319, 333, 347
Notes and Queries, 24, 38, 49, 63, 76, 89, 102, 115, 128, 143, 158, 170, 183, 196, 208, 221, 236, 249, 262, 277, 290, 303, 318, 331, 346, 359
Notes on Rectal Nursing, 298, 315
Novelties for Nurses, 20, 272
Nurse in Fiction, The, 85
Nurse at the Red Cross Depôt, A, 294
Nurses' Bookshelf, The, 21, 44, 74, 85, 113, 126, 140, 259, 273, 299, 342
Nurses of the Imperial Yeomanry Hospital, The, 19, 31, 43, 71, 110
Nurses of the Indian Army, The, 11
Nurses of the Kent and Canterbury Hospital, The, 230
Nurses of the New Bethnal Green Infirmary, 9
Nurses of Plymouth Infirmary, The, 123
Nurses' Reading Society, 338
"Nurses' Settlement" in New York, A, 182
Nurses Wanted in Western Australia, 353
Nurses of the Westminster Hospital, The, 149
Nursing in America, 151, 163, 180, 191, 205
Nursing in America, A Rejoinder, 268, 284
Nursing Arrangements of Military Hospitals, The, 282
Nursing on Board the "Assaye," 355
Nursing on Board the "Orcana," 267
Nursing in the Civil Hospital, St. Helena, 121
Nursing in a Colliery District, 315
Nursing of Dysentery, The, 16, 34
Nursing in a Field Hospital in Natal, 167
Nursing at the General Hospital, Trieste, 269
Nursing at the German Hospital, 69
Nursing at the Imperial Yeomanry Hospital, 135
Nursing at the Jenny Lind Infirmary for Sick Children, Norwich, 175
Nursing at Kimberley and on Board the "Britannic," 241
Nursing in Ladysmith, 133
Nursing in Las Palmas, 84
Nursing at Maritzburg, 43, 231
Nursing at a Newcastle Stationary Hospital, 324
"Nursing Notes" Exhibition at Earl's Court, 113
Nursing at No. 6 General Hospital, Johannesburg, 339, 353
Nursing in the Open Air, 340
Nursing on the "Orotava," 309
Nursing of Orthopædic Cases, 98, 165
Nursing of Paupers, The, 243, 274
Nursing in Sanatoria for Consumption, 215, 233, 242
Nursing the Sick and Wounded in China, 338
Nursing of the Sick Soldiers in South Africa, The, 177
Nursing at the Siege of Mafeking, 213
Nursing in the Straits Settlements, 295
Nursing in Tokio: A Difficulty, 247
Nursing in Tubercular Disease of the Joints, 111, 136
Nursing the Wounded on the "Austral," 70
Nursing the Wounded at Naauwpoort, 17, 152
Nursing Sister's Visit to Ladysmith, A, 123
Only Private Hospital in England, The, 5
Picnic for Sick Children, A, 341
Picture in Outline, A, 87
Pictures from Italy, 354
Plague Nursing in the Up Country, 274
Practical Hints for Nurses, 8
Presentations, 10, 32, 45, 57, 84, 122, 136, 164, 179, 202, 228, 244, 293, 314
Prevention of Tuberculosis, The, 168
Principles of Antiseptic Surgery and the Preparation of Patients for Operation, The, 217, 232
Princess of Wales's and Other Nurses for South Africa, 202
Private Military Hospital in England, The Only, 5
Private Monthly Nursing, 12
Private Nursing in the "Golden West," 337
Private Nursing in Rhodesia, 286
Properties of Leben, The, 154
Queen and the Dublin Nurses, The, 56
Queen's Nurses, The, 176
Queen Victoria's Jubilee Institute for Nurses, 193
Reading to the Sick, For, 23, 38, 49, 63, 76, 89, 102, 115, 128, 142, 158, 170, 183, 196, 208, 221, 236, 249, 262, 275, 290, 303, 318, 331, 346, 358
Receptions to Liverpool Nurses, 75
Remedy for Sleeplessness, 10
Robbing a Nurse in the East End, 81
Royal British Nurses' Association, 72, 178, 220
Royal Devon and Exeter Hospital, Nurses of the, 351
Royal Orthopædic Hospital, 19
St. Andrew's Home Club for Nurses, The, 258
Scenes in a French Hospital, 58
Sick and Wounded on the "Montfort," The, 192
Six Months' Nursing at the Princess Christian Hospital, 323
Some Difficulties of a Matron's Life, 137
Some of My Experiences as a Dispenser, 312
Steevens' Hospital, Dublin, Dr., 255
Tending a Shipwrecked Crew, 10
That "Pesky" Mosquito, 83
To Nurses, 17, 31, 48, 59, 87, 97, 113, 121
Travel Notes, 50, 64, 90, 116, 144, 184, 222, 250, 278, 304, 332, 360
Treatment of Burns, The, 150
Trooping the Colour, 127
Typical Patients, 301
Typhoid Nursing, Important Points in, 14
U.S.A. Hospital Ship "Relief," The, 214
Visit to Colesberg, A, 269
War in South Africa, The, 108
"Wee Donald," 344
Week in Paris, A, 154
Which? 150

Printed by W. H. and L. Collingridge, City Press, 148 and 149, Aldersgate Street, London.

**IMPORTANT NOTICE.**

On account of the Easter Holidays, we beg to announce that we shall go to press with our issue of the 14th APRIL on TUESDAY NIGHT, the 10th inst. Advertisements intended for insertion in this issue should therefore reach us by 4 p.m. ON TUESDAY, the 10th inst.

### NOTICE TO CORRESPONDENTS.

All MS., Letters, Books for Review, and other matters intended for the Editor should be addressed The Editor, "The Hospital," The Hospital Building, 28 & 29, Southampton Street, Strand, W.C.

The Editor cannot undertake to return rejected MS., even when accompanied by stamped directed envelope.

**Notice to Advertisers and Subscribers.**

All Advertisements, Orders for Copies of The Hospital, and Business Communications should be addressed to **THE MANAGER (Not to the Editor)**, The Hospital, The Hospital Building, 28 & 29, Southampton Street, Strand, London, W.C.

The Cost of Subscription, post free, is as follows:—Per week, 2½d.; per quarter, 2s. 8d.; per half-year, 5s. 3d.; per annum, 10s. 6d. Foreign:—Per annum, 15s. 2d., payable, in all cases, in advance.

**NOTICE.—Vol. XXVI. of "The Hospital" (April, 1899, to September, 1899), in handsome cloth binding, is now on sale at the Office of this Journal, price 7s. 6d. Cases for binding the Numbers contained in this Volume 1s. 6d. Index to Vol. XXVI., 2½d., post free.**

**The Monthly Part for April is now ready. Price 6d., by post 8d.**

### Notes and News.

### BOOKS RECEIVED.

H. K. Lewis.

"A Manual of Gynæcological Practice." By D. A. Dührssen. Translated from the Sixth German Edition by John W. Taylor, F.R.C.S., and Frederick Edge, M.D. Lond., M.R.C.P., F.R.C.S.

"Anæsthetics: Their Uses and Administration." By Dudley Wilmot Buxton, M.D., B.S. Third edition.

J. and A. Churchill.

"The Student's Handbook of the Surgery of the Alimentary Canal." By A. Ernest Maylard, M.B., B.S.Lond.

### INSTITUTIONAL QUESTIONS.

### THE STUDENT OF MEDICINE.

**APPOINTMENTS.**

H. P. Ainsworth, L.R.C.P.Lond., M.R.C.S.Eng., appointed Medical Officer for the Fourth District of the North Witchford Union. H. N. Baron, L.R.C.P.Lond., M.R.C.S.Eng., appointed Medical Officer for the Orford District of the Plomesgate Union. G. T. Birkett, L.R.C.P., L.R.C.S.Edin., appointed Medical Officer for the Catford District of the Lewisham Union. G. Broadbent, L.R.C.P., L.R.C.S., appointed Medical Officer for the Maltby District of the Rotherham Union. C. Brook, M.R.C.S., L.S.A., appointed Consulting Surgeon to the Lincoln County Hospital. W. H. B. Brook, M.D., B.S.Lond., F.R.C.S., appointed Surgeon to the Lincoln County Hospital. G. H. Edington, M.D., M.R.C.S., appointed Visiting Surgeon to the Glasgow Training Home for Nurses. C. Firth, M.D.Lond., F.R.C.S., appointed Medical Officer for the Children's Homes of the Gravesend and Milton Union. E. G. L. Goffe, M.B., B.S.Lond., appointed Assistant Medical Officer to the Infirmary and Workhouse of the Lewisham Union. R. Hopkin, M.R.C.S., L.R.C.P.Lond., appointed Medical Officer for the Second District of the Llandovery Union. D. W. Jones, L.R.C.P., L.R.C.S.Edin., appointed Medical Officer for the Rotherham District of the Rotherham Union. G. Lapraik, M.B., C.M.Glasg., appointed Public Vaccinator for the District of Thames, New Zealand. T. C. Lawson, M.R.C.S.Eng., appointed Medical Officer for the Longtown District of the Dore Union. J. McMurray, M.D., appointed Medical Officer for the Kirkdale Branch Workhouse of the Parish of Liverpool. A. P. Macnamara, M.B., appointed Medical Officer of Health for the Shrivenham District of the Faringdon Union. [illegible] B. Mahon, M.D.R.U.I., F.R.C.S.Eng., appointed Certifying Factory Surgeon for the Ballinrobe Dispensary District, co. Mayo. E. H. L. Oliphant, M.D.Edin., F.F.P.S.G., appointed Physician to the Dispensary for Diseases of Women, Glasgow Western Infirmary. H. A. Pearson, L.R.C.P., L.R.C.S., appointed Medical Officer for the Fifth District of the Royston Union. W. L. Rhys, L.R.C.P.Lond., M.R.C.S.Eng., appointed Medical Officer of the Aberdare Town District.

Royal 8vo., illustrated by a series of Original Photo-micrographs and many Illustrations in the Text, cloth gilt, 7s. 6d. net,

**THE MENOPAUSE AND ITS DISORDERS.**

**With Chapters on Menstruation.**

By A. D. LEITH NAPIER, M.D., M.R.C.P.

London: The Scientific Press, Ltd., 28 & 29, Southampton Street, Strand, W.C.

"The Prototype of all Bitter Waters."—Lancet.

### Hunyadi János

"Speedy, sure and gentle."—Brit. Med. Jour., 1884.
"Speedy, sure and gentle."—Brit. Med. Jour., 1890.
"Speedy, sure and gentle."—Practitioner, 1896.

PROPRIETOR:

ANDREAS SAXLEHNER, BUDAPEST.

### ROYAL NATIONAL PENSION FUND FOR NURSES,

28, FINSBURY PAVEMENT, LONDON, E.C.

### PENSIONS—SICKNESS—ACCIDENT.

**President—H.R.H. THE PRINCESS OF WALES.**

THE COUNCIL.

Everard A. Hambro, Esq. (Messrs. C. I. Hambro and Son), Chairman.
Sir Henry Burdett, K.C.B. (the Founder), Deputy-Chairman.
Sir William Broadbent, Bart., M.D., F.R.S., F.R.C.P., Consulting Physician, St. Mary's Hospital.
Thos. Bryant, Esq., F.R.C.S., Consulting Surgeon, Guy's Hospital.
Walter S. M. Burns, Esq. (Messrs. J. S. Morgan and Co.)
C. Eric Hambro, Esq. (Messrs. C. I. Hambro and Sons).
Herbert P. Hawkins, Esq., M D., F.R.C.P., Physician, St. Thomas's Hospital
The Hon Egremont J. Mills (Messrs. Marks, Bulteel, and Co.)
J. Pierpont Morgan, jun, Esq. (Messrs. J. S. Morgan and Co.)
Arthur H. A. Morton, Esq., M.P., Athenæum Club, Pall Mall, S.W.
G. Norman, Esq., Oakley, Bromley, Kent.
E. C. Perry, Esq., M.D., F.R.C.P., Physician and Superintendent, Guy's Hospital.
Edward Rawlings, Esq. (late of Messrs. C. I. Hambro and Son).
Alfred Charles de Rothschild, Esq. (Messrs. N. M. Rothschild and Sons).
The Hon. Walter Rothschild, M.P. (Messrs. N. M. Rothschild and Sons).
Charles W. Trotter, Esq. (Messrs. James Capel and Co.)
John Watney, Esq. (Mercers' Hall, E.C.)

**Bankers**—The Bank of England.
**Honorary Counsel**—Harry T. Eve, Esq., Q.C., 4, New Square, W.C.
**Consulting Actuary**—George King, F.I.A., F.F.A. **Medical Referee**—Geo. W. Potter, M.D.
**Auditor**—Frederick Whinney, F.C.A., 32, Old Jewry, E.C.
**Solicitor**—Perceval A. Nairne, 3, Crosby Square, E.C.
**Secretary**—Louis H. M. Dick.

### STATEMENT OF THE RESULTS FOR 1899.

| | | |
|---|---|---|
| New Policies issued ... | during the year ... ... | 811 |
| Premiums received ... ... | ditto ... ... ... | £68,210 |
| Pensions paid ... ... ... | ditto ... ... ... | £3,566 |
| Sick pay distributed to Policyholders, | ditto ... ... ... | £1,296 |

### INVESTED FUNDS
### HALF A MILLION STERLING.

OVER **£8,300** HAS BEEN DISTRIBUTED IN SICK PAY.

**More than £70,000 has been returned** to Nurses who have been obliged to give up their Policies from one cause or another.

**THE ABOVE FIGURES SPEAK FOR THEMSELVES AS REGARDS THE APPRECIATION IN WHICH THE FUND IS HELD BY THOSE FOR WHOM IT WAS INSTITUTED.**

Full particulars to be obtained from the SECRETARY,

ROYAL NATIONAL PENSION FUND FOR NURSES,

28, FINSBURY PAVEMENT, LONDON, E.C

